# Autism spectrum disorder and genetic: a possible correlation?

**DOI:** 10.1192/j.eurpsy.2023.1881

**Published:** 2023-07-19

**Authors:** M. Violi, M. Simoncini, A. Valetto, V. Bertini, F. Pardini, L. Massoni, C. Carmassi, L. Dell’Osso

**Affiliations:** 1 DEPARTEMENT OF CLINICAL AND EXPERIMENTAL MEDICINE; 2 Departmental Section of Cytogenetics, UNIVERSITY OF PISA, PISA, Italy

## Abstract

**Introduction:**

Autism spectrum disorder (ASD) is a neurodevelopmental disorder characterized by impairment in social interaction and communication, whose etiology is heterogeneous, including genetic, epigenetic, and environmental factors. It is associated with restricted interests and stereotyped behaviors with high prevalence rates in general population, neurobiological bases and high heritability.

**Objectives:**

The aim of our study is to identify the possible phenotype-genotype relationships regarding neurodevelopmental disorders and to evaluate a correlation between genomic alterations and the manifestations of the overt and subthreshold ASD in a family administered psychiatric clinical evaluations at our hospital.

**Methods:**

The family M underwent a psychiatric evaluation through the MINI interview according to the SCID-5 criteria, the AQ, ADAS, PAS-SR, SHI-SHY,SHY-OBS to assess respectively the subthreshold traits of ASD in adulthood, the panic-agoraphobic, social-phobic and obsessive-compulsive spectrum and CAT-Q Italian version, to evaluate social camouflage behaviors typical of ASD individuals. Array Comparative Genomic Hybridization was used for studying DNA imbalances in this family.

**Results:**

We found that Mrs.A, her father, her brothers and her older sister had a microduplication, very likely pathogenic, since it has been never reported in healthy subjects and harbors several genes. It could be related with overt and subthreshold traits of ASD. From the questionnaires administered and from the clinical interview, it emerged that Mrs.A is affected by ASD and Bipolar Disorder. Her twin brothers have been evaluated at early ages by child neuropsychiatric clinic and they were diagnosed with ASD and mental and psychomotor impairment. Her father was reported a significant trend in ADAS, AQ, PAS-SR, SHI-SHY and SHY-OBS scores. Finally, about her older sister, even if our results were not significant for an ASD diagnosis, we speculated that she performed a high score in some ADAS items and in the CAT-Q but not in the AQ. Females generally tend to attract fewer attention than males thanks to their better coping and camouflaging mechanisms as well as their ability to “disappear” in large groups.

**Conclusions:**

Genetic knowledge can have a relevant clinical impact; a genetic etiology can be identified in individuals with ASD, leading to the identification of treatable psychiatric comorbidities. Furthermore, knowing the causative genetic variants of ASD could provide crucial information for genetic counseling as well as to understand the neurobiology of these disorders and to contribute to an early diagnosis.

**Disclosure of Interest:**

None Declared

